# Breast Cancer in the Presence of Failed Saline Breast Implants

**DOI:** 10.7759/cureus.14204

**Published:** 2021-03-31

**Authors:** Sherif Monib, Simon Thomson

**Affiliations:** 1 Breast Surgery, West Hertfordshire Hospitals NHS Trust, St. Albans, GBR

**Keywords:** breast cancer, breast implant, saline implant, failed implant

## Abstract

Breast augmentation has been gaining popularity over the last two decades to correct congenital breast asymmetry or increase breast size and projection. Augmentation options started with saline implants, then silicone implants, and, recently, autologous fat transfer.

Unfortunately, breast implants are not without complications, some of which are common, like capsular contracture, implant failure and infection. Others are quite rare, such as Breast Implant-Associated Anaplastic Large Cell Lymphoma (BIA-ALCL). Most of these complications will eventually require explantation in most cases, as the patients’ and implants' age and risk of complications increase.

We present a 79-year-old patient who presented to our breast unit with a left breast lump with 50-year-old saline implants. A triple assessment revealed incidental right breast cancer treated with radiofrequency identification (RFID) tag-guided wide local excision, sentinel lymph node biopsy and bilateral explantation.

## Introduction

Breast augmentation started gaining popularity in the sixties until it became the second-most popular form of cosmetic surgery worldwide. With recent advances in medical sciences and appreciation of the importance of considering patients' psychological and physical needs, different breast options for augmentation evolved to better achieve patients' satisfaction. Various types of breast implants have facilitated tailoring breast augmentation and reconstruction to the patients' needs, to achieve the desired shape, size and symmetry.

The lifetime incidence of female breast cancer is approximately 12.3%. About 48% of cancers are seen in patients over 65, and about 30% of those cases are seen in the population over 70 [1]. The incidence of breast cancer in women with implants is on the rise due to the marked increase in breast augmentation surgeries using breast implants in recent years. This group of patients' clinical management is challenging, as aesthetic results following breast-conserving surgery are likely to be compromised due to different cancer treatment modalities or implant-related complications [2].

As patients and implants age, the incidence of breast cancer and implant-related complications increases [3], so clinicians will be faced with a group of patients, who are relatively old, diagnosed with breast cancer in the presence of failing or failed implants. Unfortunately, this group of patients is not well-represented in the literature. There is no consensus on surgical management. Often, their treatment is based on the clinician's experience rather than solid guidelines.

We are presenting a case of breast cancer who had one of the very early saline breast implants for augmentation.

## Case presentation

We present a case of a 79-year-old independent female who was referred to our breast unit with a left breast lump. Her past medical history included bilateral implant-based breast augmentation in 1970, hypertension, myocardial infarction in 1979 and 1982, coronary stents, atrioventricular (AV) node ablation, and atrial fibrillation (AF). There was no relevant family history, and the general examination was unremarkable. Breast examination revealed bilateral hard 50 mm masses in each breast's centre, denoting grade IV capsular contracture (as per Baker scale [4]) and likely failed breast implants, with no other palpable suspicious lumps or axillary or supraclavicular lymphadenopathy.

**Figure 1:**
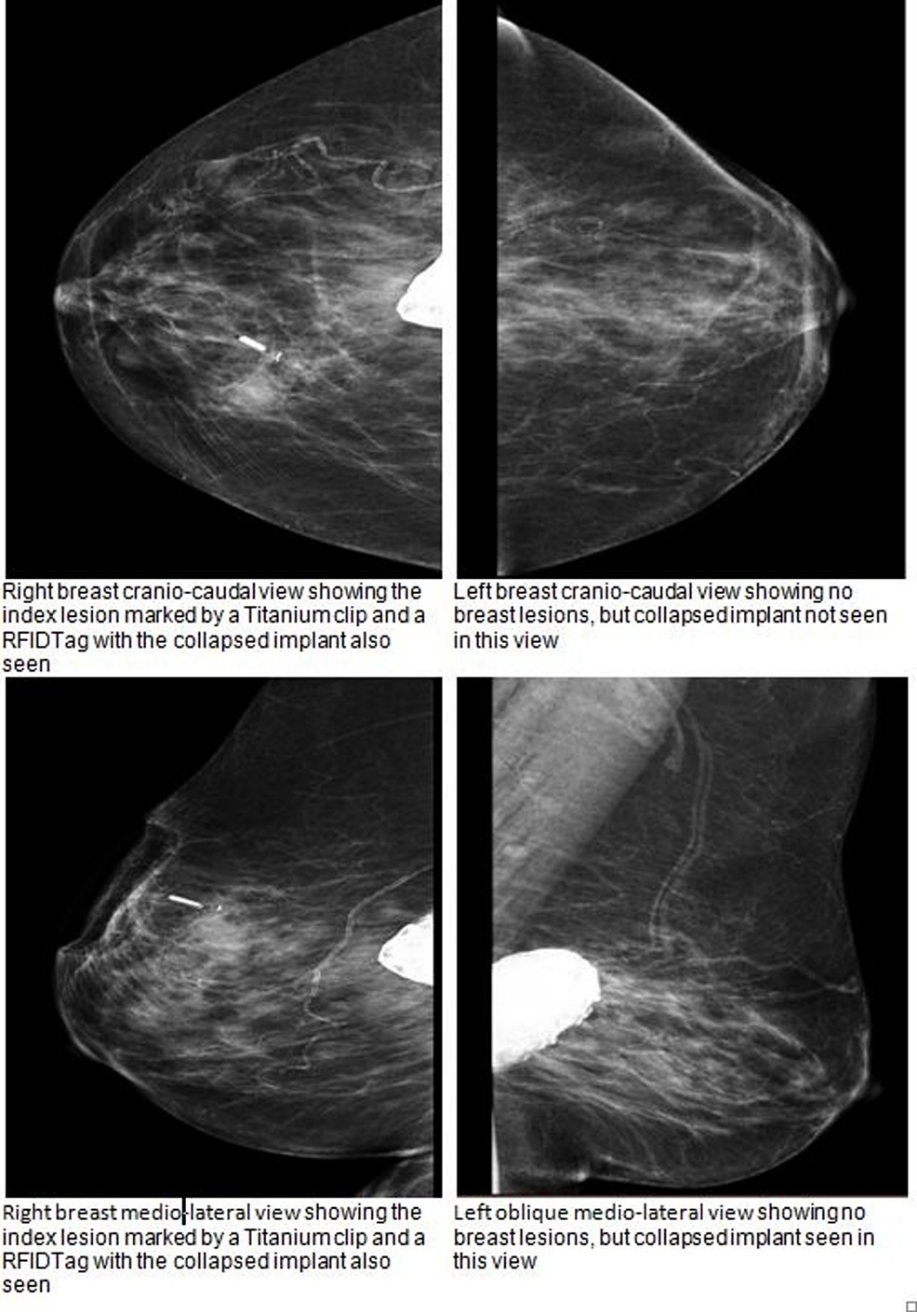
Preoperative cranio-caudal and medio-lateral mammograms showing the right breast cancer area, as well as bilateral calcified failed implants

**Figure 2:**
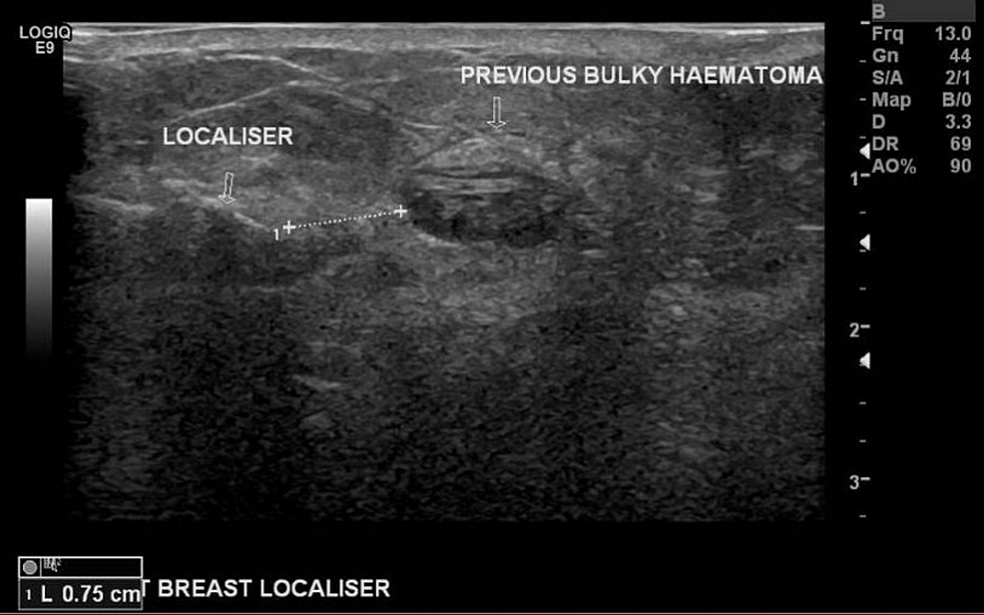
Preoperative right breast ultrasound scan showing the cancerous lesion after localization using a radiofrequency identification (RFID) tag

Bilateral mammogram and ultrasound scan showed an impalpable, right breast, 10 mm, suspicious mass, which was cored and clipped under ultrasound scan guidance. They also showed bilateral failed collapsed breast implants (Figures 1-2).

**Figure 3:**
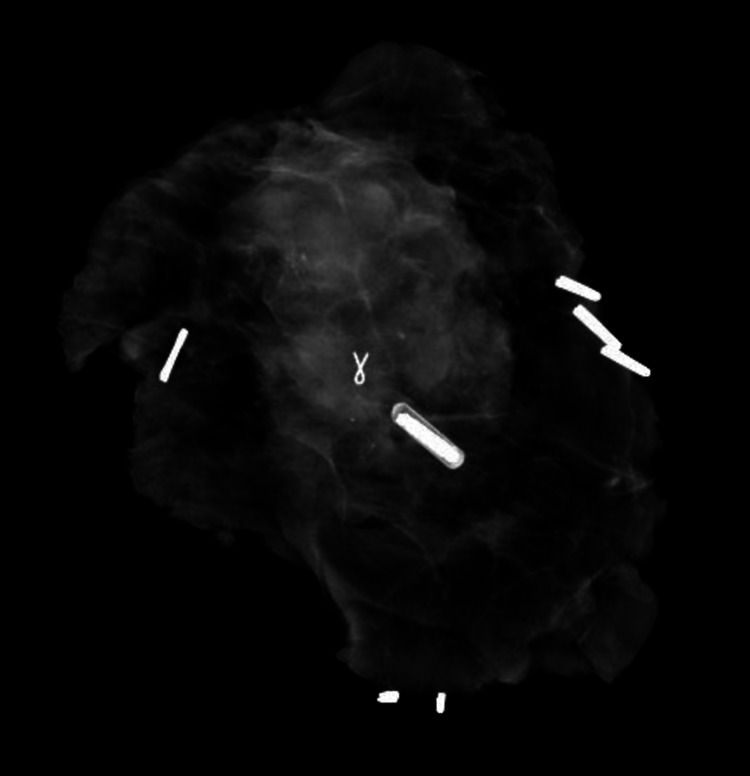
Intraoperative X-ray specimen of the right breast wide local excision specimen showing the cancerous index lesion, Titanium clip as well as the radiofrequency identification (RFID) tag

**Figure 4:**
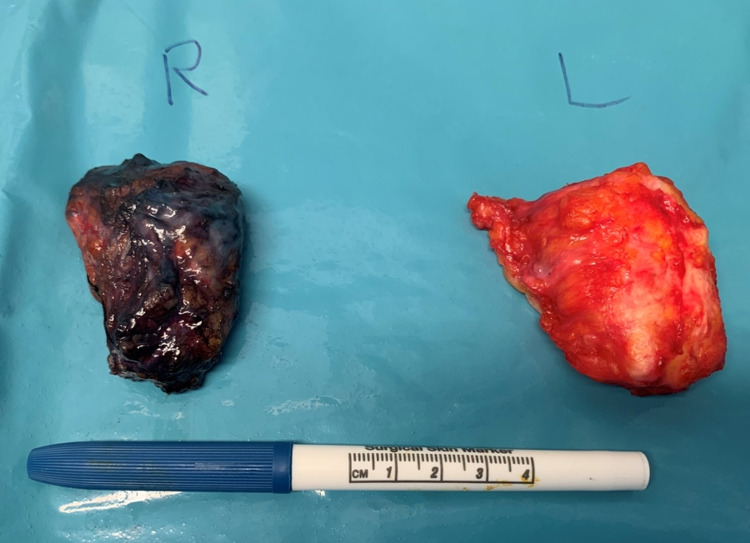
Operative specimen showing bilateral excised collapsed implant on the right side; patent blue dye stained the specimen

After discussing her care in our multidisciplinary meeting, we preceded with right breast radiofrequency identification (RFID) tag-guided wide local excision (Figure 3), patent blue and radioactive directed sentinel lymph node biopsy (SLNB) and bilateral explantation surrounded by a rim of normal breast tissue (Figure 4).

The patient had smooth postoperative recovery and was discharged the following day, seen in the clinic the following week with good aesthetic results and no postoperative complications were noted.

Final histology revealed a fully excised, right breast, 17 mm, invasive ductal carcinoma, grade II, with intermediate-grade solid and comedo ductal carcinoma in situ, estrogen receptors (ER) 8, progesterone receptors (PR) 8 and human epidermal growth factor receptor 2 (HER2) negative, and SLNB 0/2. The bilateral collapsed implants showed an extensively calcified capsule lined by a band of dense hyaline fibrous tissue showing patchy dystrophic calcification, with mild chronic inflammation, occasional foreign body giant cells, and fat necrosis noted but no evidence of Breast Implant-Associated Anaplastic Large Cell Lymphoma (BIA-ALCL). Based on the final histology, the patient was offered right breast radiotherapy, followed by endocrine treatment.

## Discussion

Before introducing silicone implants, breast augmentation options included injections of glycerine, silicone oil, autologous fat, ox cartilage and even snake venom, which were associated with a high rate of complications [5]. From 1951 to 1963, a number of different sponges were used for breast augmentation, including polyvinyl alcohol sponge and polyethylene sponge. Still, they were associated with a very high incidence of capsular contracture [6]. In 1963, Cronin and Gerow introduced the first silicone gel breast implant [7]. In 1964, saline implants were introduced by Henry Jenny and Laboratoires Arion in France as a response to refuted silicone worries [8].

In 1969, Ashley introduced anatomical shaped implants covered in polyurethane foam, which was found to have significantly reduced the high rate of capsular contracture [9]. In 1982, Radovan introduced tissue expanders [10]. In 1984, Becker described a dual-chamber expander with a silicone gel outer lumen with an inflatable inner saline lumen, which led to the possibility of having a single-stage breast reconstruction [11].

The incidence and risk factors of implant-related complications vary. Infection is seen in about 2% of patients; two-thirds develop in the early postoperative period while one-third develops years after surgery. Infection rates are known to be higher after breast reconstruction surgery, especially immediate reconstruction than after breast augmentation [12]. Capsular contracture is another well-recognised complication that can be attributed to low-grade or subclinical infection and biofilm formation [12-13].

The incidence of breast implant failure was found to increase with implant age; about 15% of implants are expected to fail between the third and tenth years [3]. Yet, in our case, the patient started to feel implant-related changes in the form of a possible lump, indicating failure about 50 years following implantation.

Breast implant failure can sometimes be complicated with infection, leading to significant morbidity and implant loss [14]. Therefore, clinicians need to stay vigilant and look out for clinical signs of implant-related complications, especially in elderly patients with breast implants.

The aesthetic outcome of implant-based breast augmentation depends not only on patients’ expectations, chest wall shape and symmetry [15], breast shape, consistency and compliance or laxity of the lipo-cutaneous envelope but also on implant shape, fill and size [16]. Hence, saline-filled implants, which were quite popular in the past, are less popular now with the recent advances in silicone implants.

While some studies have suggested that breast implants can affect the detection of breast cancer at screening settings, Azzi et al. have found that breast implant, either subpectoral or subglandular, does not impact cancer screening [17]. Other studies have also suggested that cosmetic breast augmentation can adversely affect patients' survival, who subsequently develop breast cancer. These findings should be interpreted with caution, as, unfortunately, some of these studies did not adjust for potential confounders [18].

## Conclusions

We believe we have presented one of the very early breast augmentation cases using saline implants. This patient developed breast cancer 50 years after having her implants. Luckily enough, she presented with left implant-related changes, leading to the early diagnosis of her right breast cancer. Our case highlighted the importance of history-taking and triple assessment in ruling out breast implant complications and incidental cancer, especially in geriatric patients, which will aid individualised patient management.

We recommend a meta-analysis of breast cancer patients' management with coexistent breast implants to develop more robust, evidence-based guidelines for managing this group of patients to achieve better outcomes.
